# A Case of Puerperal Convulsions

**Published:** 1807-10

**Authors:** 


### *A Case of Puerperal Convulsions.*

[ Extracted from the Philadelphia Medical Museum. ]

ON July 28th, 1799, I was requested to visit Mrs. D. aged about forty, of a full habit, labouring under puerperal convulsions. About nine, A. M. yesterday, the waters came away, and every thing went on well, though rather tedious. At nine, P. M. she was safely delivered of twins, which were small, but healthy. The placenta came away soon after, with the loss of but little blood. Her spirits were raised at the happy termination of the labour, in consequence of which she exerted herself greatly in talking with her neighbours; and got out of bed not less than three or four times during the night, to discharge her urine, which came away in large quantity. The gentleman who attended her, told me he left her last evening in the most favourable state. This morning, about nine o'clock, having slept but little, and being very restless during the night, with a slight discharge of the lochiæ, she was seized with a convulsion. Another succeeded in half an hour, and by the time I saw her at one, P. M. she had had no less than seven. She was now insensible, very restless, and frequently sighing, skin hot, pulse quick and strong, face much flushed. Her pulse this morning was very low, and she had taken assafœtida, vol. alkali, and opium, without relief. Her bowels were well opened yesterday. I ordered her to lose ten ounces of blood, which were taken away whilst I remained, with difficulty, owing to the convulsions. She bled freely, and for a short time seemed rather easier. In about ten minutes she had a fit, continuing ten or fifteen minutes, and terminating in an apoplectic stertor, with foaming at the mouth. During the fit the pulse was full and strong. I ordered an emollient glyster, with warm fomentations to the abdomen; and directions to take away ten ounces more of blood, if the pulse kept up, and the convulsions did not moderate.

At six, P. M. I found her more quiet, and sleeping easier; has had four more fits, at longer intervals; and at times she appeared to be sensible, by calling for her children, and by knowing her attendants; the skin is cooler, and is more generally moist; slight appearance of the lochiæ. The pulse kept up so much, that she lost the blood as I had directed between three and four, P. M.; pulse still strong and quick, above 140 in a minute. The injection was retained. If the symptoms do not moderate, let her her lose twelve ounces of blood in the course of the evening, and apply a large pair of blisters to the ankles. The face is less flushed, and the head appears relieved, as she has less of the stertorous breathing, and less general inquietude. The blood drawn was not sizy. I am told she is rather intemperate in health.

29th, nine, A. M. Four fits, but less violent during the night; she is more sensible, though restless; blisters rose well; lost ten ounces of blood in the evening, which is not sizy; no appearance of lochiæ; sleeps more easy. During the night her bed broke down, and she was obliged to sit up in a chair till it was mended. Passed her water several times, but has had no stool; considerable pain of abdomen, but no sickness or vomiting; breasts rather turgid; pulse still active and strong, and 130 in a minute; tongue white and much bitten during the convulsions. Change her linen, &c. daily; continue fomentations to the abdomen; bleed to ten ounces if the pulse keeps up, and let her drink punch made of cremor tartar, If her bowels are not soon open give moderate doses of soluble tartar or Glauber's salts.

Eight, P. M. I found her in a composed and sound sleep, pulse 116, and more moderate; skin cooler and less flushed; one copious stool, with great relief. At three, P. M. the pulse had risen so greatly that ten ounces of blood (not sizy) were taken away. Breasts considerably hard. Apply the children to them. Omit the cream of tartar, as she has taken about two table spoonfuls. Barley water or molasses and water for her common drink, with ℈ij. of nitre to each quart. Has had no convulsions Slight appearance of the lochial discharge. Appears more sensible than heretofore. Before the last bleeding she complained of very great sensibility of the skin; even tying up the arm to bleed gave considerable pain. This has subsided. Repeat the bleeding if the pulse rises.

30th. Bowels well opened during the night. Since when, she has been much disposed to hysteria, laughing and talking continually, except whilst sleeping, which is tolerably composed, No convulsions. Breasts more turgid. Apply the children to them. Lochiæ increase. Complains of pain in her side and back Bathe the parts with volatile liniment. No pain of abdomen. Urine natural. Pulse from 112 to 120, and more moderate. Bleeding not requisite in the night. Tongue white and moist, and much injured by biting it during the convulsions. Ordered nitre grs. x, and one-eighth of a grain of tartar tartar emetic every two hours; and if the pain of the side increases apply a blister, and bleed to six or eight ounces.

31st. Appears much better. Lochiæ in small quantities. Pain of side relieved by the embrocation. Two stools last night and one this morning with much relief. Pulse nearly 130, but moderate. Tongue white and moist. Talked a great deal yesterday, and saw too many friends. Pain of abdomen is gone. Slept but little till the morning. Continue the nitre and tartar emetic, and use gruel without wine, for nourishment.

August 1st. Slept well by the aid of ten drops of laudanum. Much better. Milk increases, and lochiæ in usual quantities. Pulse 100, and moderate. Tongue cleaner. Sat up yesterday in the afternoon. Urine frequent. Complains of her bruises. Continue nitre, &c. and take broth.

3d. Convalescent. In less than a week from this period she was down stairs.

